# Better duplicate detection for systematic reviewers: evaluation of Systematic Review Assistant-Deduplication Module

**DOI:** 10.1186/2046-4053-4-6

**Published:** 2015-01-14

**Authors:** John Rathbone, Matt Carter, Tammy Hoffmann, Paul Glasziou

**Affiliations:** Centre for Research in Evidence Based Practice, Bond University, Gold Coast, Australia

**Keywords:** Systematic Review Assistant-Deduplication Module, SRA-DM, Deduplication, EndNote, Reference-manager, Citation-screening, Systematic review, Bibliographic database

## Abstract

**Background:**

A major problem arising from searching across bibliographic databases is the retrieval of duplicate citations. Removing such duplicates is an essential task to ensure systematic reviewers do not waste time screening the same citation multiple times. Although reference management software use algorithms to remove duplicate records, this is only partially successful and necessitates removing the remaining duplicates manually. This time-consuming task leads to wasted resources. We sought to evaluate the effectiveness of a newly developed deduplication program against EndNote.

**Methods:**

A literature search of 1,988 citations was manually inspected and duplicate citations identified and coded to create a benchmark dataset. The Systematic Review Assistant-Deduplication Module (SRA-DM) was iteratively developed and tested using the benchmark dataset and compared with EndNote’s default one step auto-deduplication process matching on (‘author’, ‘year’, ‘title’). The accuracy of deduplication was reported by calculating the sensitivity and specificity. Further validation tests, with three additional benchmarked literature searches comprising a total of 4,563 citations were performed to determine the reliability of the SRA-DM algorithm.

**Results:**

The sensitivity (84%) and specificity (100%) of the SRA-DM was superior to EndNote (sensitivity 51%, specificity 99.83%). Validation testing on three additional biomedical literature searches demonstrated that SRA-DM consistently achieved higher sensitivity than EndNote (90% vs 63%), (84% vs 73%) and (84% vs 64%). Furthermore, the specificity of SRA-DM was 100%, whereas the specificity of EndNote was imperfect (average 99.75%) with some unique records wrongly assigned as duplicates. Overall, there was a 42.86% increase in the number of duplicates records detected with SRA-DM compared with EndNote auto-deduplication.

**Conclusions:**

The Systematic Review Assistant-Deduplication Module offers users a reliable program to remove duplicate records with greater sensitivity and specificity than EndNote. This application will save researchers and information specialists time and avoid research waste. The deduplication program is freely available online.

## Background

Identifying trials for systematic reviews is time consuming: the average retrieval from a PubMed search produces 17,284 citations [1]. The biomedical databases MEDLINE [2] and EMBASE [3] contain over 41 million records, and about one million records are added annually to EMBASE [3] (which now also includes MEDLINE records) and 700,000 to MEDLINE [2]. However, the methodological details of trials are often inadequately described by authors in the titles or abstracts, and not all records contain an abstract [4]. Due to these limitations, a wider (that is, more sensitive) search strategy is necessary to ensure articles are not missed, which leads to an imprecise dataset retrieved from electronic bibliographic databases. Typically of the thousands of citations retrieved for a systematic review search, over 90% are excluded on the basis of title and abstract screening [5].

Searching multiple databases is essential because different databases contain different records, and therefore, the coverage is widened. Also, searching multiple databases utilises differences in indexing to increase the likelihood of retrieving relevant items that are listed in several databases [6], but inevitably, this practice also retrieves overlapping content [7]. The degree of journal overlap estimated by Smith [8] over a decade ago indicated that about 35% of journals were listed in both MEDLINE and EMBASE. Journal overlap can vary from 10% to 75% [9, 10, 8, 11, 12] depending on medical speciality. More recently, the overlap in MEDLINE and EMBASE was found to be 79% [13] based on trials that had been included in 66 Cochrane systematic reviews.

The problem of overlapping content and subsequent retrieval of duplicate records is partially managed with commercial reference management software programs such as EndNote [14], Reference Manager [15], Mendeley [16] and RefWorks [17]. They contain algorithms designed to identify and remove duplicate records using an auto-deduplication function. However, the detection of duplicate records can be thwarted by inconsistent citation details, missing information or errors in the records. Typically, auto-deduplication is only partially successful [18], and the onerous task of manually sifting and removing the remaining duplicates rests with reviewers or information specialists. Removing such duplicates is an essential task to ensure systematic reviewers do not waste time screening the same citation multiple times. This study aimed to iteratively develop and test the performance of a new deduplication program against EndNote X6.

## Methods

### Systematic Review Assistant-Deduplication Module process of development

The Systematic Review Assistant-Deduplication Module (SRA-DM) project was developed in 2013 at the Bond University Centre for Research in Evidence-Based Practice (CREBP). The project aimed to reduce the amount of time taken to produce systematic reviews by maximising the efficiency of the various review stages such as optimising search strategies and screening, finding full text articles and removing duplicate citations.

The deduplication algorithm was developed using a heuristic-based approach with the aim of increasing the retrieval of duplicate records and minimising unique records being erroneously designated as duplicates. The algorithm was developed iteratively with each version tested against a benchmark dataset of 1,988 citations. Modifications were made to the algorithm to overcome errors in duplicate detection (Table 1). For example, errors often occurred due to variations in author names (e.g. first-name/surname sequence, use/absence of initialisation, missing author names and typographical errors), page numbers (e.g. full/truncated, or missing), text accent marks (e.g. French/German/Spanish) and journal names (e.g. abbreviated/complete, and ‘*the*’ used intermittently). The performance of the SRA-DM algorithm was compared with EndNote’s default one step auto-deduplication process. To determine the reliability of SRA-DM, we conducted a series of validation tests with results of different literature searches (cytology screening tests, stroke and haematology) which were retrieved from searching multiple biomedical databases (Table 2).

**Table 1:** **SRA-DM algorithm changes**

Iterations	Changes to algorithms
First iteration	Matching criteria were based on simple field comparison (ignoring punctuation) with checks against the year field since this field has a lower probability for errors because it is restricted to integers 0–9 and therefore the best non-mistakable field.
Second iteration	Short format page numbers were converted to full format (e.g. 221–226, 221–6), and the algorithm was further modified to increase the sensitivity by incorporating matching criteria on authors OR title.
Third iteration	Match author AND title with the extension of the non-reference fields from only ‘year’ to year OR volume OR edition.
Fourth iteration	The fourth algorithm extended the matching criteria of the third algorithm, with the addition of an improved name matching system. This was context aware of author name variations, i.e. initialisation, punctuation and rearranged author listings using fuzzy logic, so that differences could be accommodated. For example, the following names are all syntactically equivalent and will match as identical authors:
	1. William Shakespeare
	2. W. Shakespeare
	3. W Shakespeare
	4. William John Shakespeare
	5. William J. Shakespeare
	6. W. J. Shakespeare
	7. W J Shakespeare
	8. Shakespeare, William
	9. Shakespeare, W
	10. Shakespeare, W, A
	11. Shakespeare, W, A, B, C
	12. William Shakespeare 1st
	13. William Shakespeare 2nd
	14. William Shakespeare IV
	15. William Adam Bob Charles Shakespeare XVI

**Table 2:** **Databases searched for retrieval of citations for validation testing**

Datasets	Databases searched
**Cytology screening tests dataset**	1. Cochrane Controlled Trials Register (CCTR)
	2. Cochrane Database of Systematic Reviews (CDSR)
	3. EMBASE
	4. MEDLINE
	5. National Research Register (NRR)
	6. Database of Assessments of Reviews of Effectiveness (DARE)
	7. NHS Health Technology Assessment (HTA)
	8. PreMEDLINE
	9. Science Citation Index
	10. Social Sciences Citation Index
**Haematology dataset**	1. MEDLINE
	2. EMBASE
	3. MEDLINE In-Process
	4. Biological Abstracts
	5. NHS Health Technology Assessment (HTA)
	6. Cochrane Controlled Trials Register (CCTR)
	7. Cochrane Database of Systematic Reviews (CDSR)
	8. CINAHL
	9. Science Citation Index
	10. Social Sciences Citation Index
**Stroke dataset**	1. MEDLINE
	2. EMBASE
	3. CENTRAL
	4. CINAHL
	5. PsycInfo

#### Definitions

A duplicate record was defined as being the same bibliographic record (irrespective of how the citation details were reported, e.g. variations in page numbers, author details, accents used or abridged titles). Where further reports from a single study were published, these were not classed as duplicates as they are multiple reports which can appear across or within journals. Similarly, where the same study was reported in both journal and conference proceedings, these were treated as separate bibliographic records.

#### Testing against benchmark

A total of 1,988 citations, derived from a search conducted on 29 July 2013 for surgical and non-surgical management for pleural empyema were used to test SRA-DM and EndNote X6. Six databases were searched (MEDLINE-Ovid, EMBASE-Elsevier, CENTRAL-Cochrane library, CINAHL-Ebasco, LILACS-Bireme, PubMed-NLM). To create the benchmark, citations were imported into EndNote database, sorted by author, inspected for duplicate records and manually coded as a unique or duplicate record; the database was reordered by article title and reinspected for further duplicates. Once the benchmark was finalised, duplicates were sought in EndNote using the default one-step auto-deduplication process which used the matching criteria of ‘author’, ‘year’ and ‘title’ (with the ‘ignore spacing and punctuation’ box ticked). A few additional duplicates were identified in EndNote and SRA-DM whilst cross-checking against the benchmark decisions, and the benchmark and results were updated to take account of these.

#### Data analysis

The accuracy of the results were coded against the benchmark according to whether it was a true positive (true duplicate, i.e. correctly identified duplicate), false positive (false duplicate, i.e. incorrectly identified as duplicate), true negative (unique record) or false negative (true duplicate, i.e. incorrectly identified as unique record). This process was repeated for results received after using the SRA-DM. Sensitivity is defined as the ability to correctly classify a record as duplicate and is the proportion of true positive records over the total number of records identified as true positive and false negative. Specificity is defined as the ability to correctly classify a record as being unique or non-duplicate and is the proportion of true negative records over the total number of records identified as true negative and false positive.

## Results

### Training and development of SRA-DM

#### First and second iteration

The first iteration of the deduplication algorithm achieved 75.0% sensitivity and 99.9% specificity (Table 3). The matching criteria were based on field comparison (ignoring punctuation) with checks made against the year field. This field was chosen because the year field has a lower probability for errors since it is restricted to integers 0–9 and therefore is the best non mistakable field. Eighty-four percent of undetected duplicates arose due to variations in pages numbers (e.g. 221–226, 221–6). To address this, short format page numbers were converted to full format and the algorithm was further modified to increase the sensitivity by incorporating matching criteria on authors OR title. This increased the sensitivity of the second iteration to 95.7% with more duplicates detected, but as a consequence the number of false positives also increased (specificity 99.8%).

**Table 3:** **Sensitivity† and specificity‡ of SRA-DM prototype algorithms and EndNote auto-deduplication (in a dataset of 1,988 citations, including 799 duplicates)**

	Respiratory study				
	First iteration SRA-DM	Second iteration SRA-DM	Third iteration SRA-DM	Fourth iteration SRA-DM	EndNote
True positive (*n*) (correctly identified duplicates)	600	765	543	674	410
False negative (*n*) (duplicates missed)	199	34	256	125	391
Sensitivity (%)	*75.1*	*95.7*	*68.0*	*84.4*	*51.2*
True negative (*n*) (correctly identified unique records)	1,188	1,186	1,189	1,189	1,185
False positive (*n*) (incorrectly identified as duplicates)	1	3	0	0	2
Specificity (%)	*99.9*	*99.8*	*100.0*	*100.0*	*99.8*

#### Third iteration

The third iteration was modified to match author AND title with the extension of the non-reference fields from only ‘year’ to year OR volume OR edition. This distinguished references that were similar (e.g. same author and title combination) but contained different source publications, and this improved the specificity to 100% but the sensitivity was reduced (68.0%).

#### Fourth iteration

The fourth iteration was modified to accommodate author name variations using fuzzy logic so that differences in names spelt in full or initialised, differences in the ordering of name and different punctuation could be accommodated (Table 1); this increased the sensitivity to 84.4% by correctly identifying 674 citations as duplicates (TP), 1,189 citations as unique records (TN), no false positives occurred (100% specificity) and only 125 duplicate records were undetected (FN). This fourth iteration of SRA-DM was then compared against EndNote. EndNote identified 412 of the 1,988 citations as duplicates. Of these, 410 were correctly identified as duplicates (TP) and two were incorrectly designated as duplicates (FP), and 1,185 citations were correctly identified as unique records (TN) and 391 duplicate citations were undetected (FN). The sensitivity of EndNote was 51.2% and specificity 99.8%. Compared with EndNote, SRA-DM produced a 64% increase in sensitivity and no loss of specificity.

### Validation results

The fourth iteration of SRA-DM was further tested with three additional datasets using search topics from cytology screening tests (*n* = 1,856), stroke (*n* = 1,292) and haematology (*n* = 1,415) (Table 2). These were obtained from existing searches performed by information specialists to widen the scope of the validation tests. SRA-DM algorithm was consistently more sensitive (Table 4) at detecting duplicates than EndNote [cytology screening: 90% vs 63%; stroke: 84% vs 73% and haematology: 84% vs 64%] and specificity of SRA-DM was 100% accurate, i.e. no false positives occurred. In contrast, the average specificity of EndNote was lower (99.7). These false positives occurred in EndNote due to citations with the same authors and title being published in other journals or as conference proceeding. Compared with EndNote, the average percentage increase in duplicates detected by SRA-DM across all four bibliographic searches was 42.8%.

**Table 4:** **Sensitivity† and specificity‡ of SRA-DM and EndNote auto-deduplication (validation testing)**

	Cytology screening		Stroke		Haematology	
	SRA-DM	EndNote	SRA-DM	EndNote	SRA-DM	EndNote
True positive (correctly identified duplicates)	1,265	885	426	372	208	159
False negative (duplicates missed)	139	518	81	134	38	87
Sensitivity (%)	*90.10*	*63.08*	*84.02*	*73.52*	*84.55*	*64.63*
True negative (correctly identified unique records)	452	452	785	784	1,169	1,165
False positive (incorrectly identified duplicates)	0	1	0	2	0	4
Specificity (%)	*100.00*	*99.78*	*100.00*	*99.75*	*100.00*	*99.66*

## Discussion

Our findings demonstrated that SRA-DM identifies substantially more duplicate citations than EndNote and has greater sensitivity [(84% vs 51%), (90% vs 63%), (84% vs 73%), (84 vs 64%)]. The specificity of SRA-DM was 100% with no false positives, whereas the specificity of EndNote was imperfect.

Waste in research occurs for several methodological, legislative and reporting reasons [19–22]. Another form of waste is inefficient labouring, in part, as a consequence of non-standardised citations details across bibliographic databases, perfunctory error checking and absence of a unique trial identification number for it and its associated further multiple reports. If these issues were solved at source, manual duplicate checking would be unnecessary. Until these issues are resolved, deploying the SRA-DM will save information specialists and reviewers valuable time by identifying on average a further 42.86% of duplicate records.

Several citations were wrongly designated as duplicates by EndNote auto-deduplication due to different citations sharing the same authors and title but published in other journals or as conference proceedings. In a recent study by Jiang [23], the authors also found that EndNote, for the same reason, had erroneously assigned unique records as duplicates. It is probable that in most scenarios no important loss of data would occur; although sometimes additional methodological or outcome data are reported, and ideally these need to be retained for inspection. A recent study by Qi [18] examined the content of undetected duplicate records in EndNote and found that errors often occurred due to missing or wrong data in the fields, especially for records retrieved from EMBASE database. This also affected the sensitivity of SRA-DM, with duplicates undetected due to missing or wrong or extraneous data in the fields.

During the training and development stage, the four iterations of SRA-DM achieved sensitivities ranging from 68%, 75%, 84% and 96% with the most sensitive (96%) achieved with a trade-off in specificity (99.75%) with three false positives. For systematic reviews and Health Technology Assessment reports, the aim is to conduct comprehensive searches to ensure all relevant trials are identified [24]; thus, losing even three citations is undesirable. Therefore, the final algorithm (fourth iteration) with the lower sensitivity (84%) but perfect (100%) specificity was preferred. Future developments with SRA-DM may incorporate two algorithms, first using the 100% specific algorithm to automatically remove duplicates and another algorithm with higher sensitivity (albeit with lower specificity) to identify the remaining duplicates for manual verification. If this strategy was implemented on the respiratory dataset using the fourth and second algorithm (Table 3), only 91 out of 1,988 citations would have to be manually checked and only 34 duplicates would remain undetected.

In spite of this major improvement with the SRA-DM, no software can currently detect all duplicate records, and the perfect uncluttered dataset remains elusive. Undetected duplicates in SRA-DM occurred due to discrepancies such as missing page numbers or too much variance with author names. Duplicates were also missed because the OVID MEDLINE platform inserted additional extraneous information into the title field (e.g. [Review] [72 refs]) whereas the same article retrieved from EMBASE or other non-OVID MEDLINE platforms (i.e. PubMed, Web of Knowledge) report only the title. Some of these problems could be overcome in the future with record linkage and citation enrichment techniques to populate blank fields with meta-data to increase the detection rate.

### Strengths and weaknesses

The deduplication program was developed to identify duplicate citations from biomedical databases and has not been tested on other bibliographic records such as books and governmental reports and therefore may not perform as well with other bibliographies. However, the deduplication program was developed iteratively to remove problems of false positives and was tested on four different datasets which included comprehensive searches using 14 different databases that are used by information specialists, and therefore, similar efficiencies should occur in other medical specialities. Also, the accuracy of SRA-DM was consistently higher than that of EndNote, and these finding are probably generalizable to other biomedical database searches due to the same records types and fields used. It is possible that some duplicates were not detected during the manual benchmarking process, although the database was screened twice first by author and then by title, and additional cross-checking was performed by manually comparing the benchmark against EndNote auto-deduplication and SRA-DM decisions—thus minimising the possibility of undetected duplicates.

Whilst we compared SRA-DM against the typical default EndNote deduplication setting, we recognise that some information specialists adopt additional steps whilst performing deduplication in EndNote. For example, they may employ multi-stage screening or attempt to replace incomplete citations by updating citation fields with the ‘Find References Update’ feature in EndNote. However, many researchers and information specialists do not employ such techniques, and our aim was to address deduplication with an automated algorithm and compare it against the default deduplication process in EndNote. Qi [18] recommended employing a two-step strategy to address the problem of undetected duplicates by first performing auto-deduplication in EndNote followed by manual hand screening to identify remaining duplicates. This basic strategy is used by some information specialists and systematic reviewers but is inefficient due to the large proportion of unidentified duplicates. Other more complex multi-stage screening strategies have been suggested [25] but are EndNote-specific and not viable for other reference management software.

## Conclusions

The deduplication algorithm has greater sensitivity and specificity than EndNote. Reviewers and information specialists incorporating SRA-DM into their research procedures will save valuable time and reduce resource waste. The algorithm is open source [26] and the SRA-DM program is freely available to users online [27]. It allows similar file manipulation to EndNote and currently accepts XML, RIS and CVS file formats enabling citations to be exported directly to RevMan software. It has the option of automatic duplicate removal or manual pair-wise duplicate screening performed individually or with a co-reviewer.
